# Examining the Impact of a Public Health Message on Fish Consumption in Bermuda

**DOI:** 10.1371/journal.pone.0139459

**Published:** 2015-10-01

**Authors:** Catherine McLean Pirkle, Cheryl Peek-Ball, Eugene Outerbridge, Philippe Max Rouja

**Affiliations:** 1 Office of Public Health Studies. University of Hawai’i at Mãnoa. Honolulu, Hawai’i, United States of America; 2 Department of Health. Hamilton, Bermuda; 3 King Edward VII Memorial Hospital, Hamilton, Bermuda; 4 Department of Conservation Services. Hamilton Parish, Bermuda; Institute for Health & the Environment, UNITED STATES

## Abstract

**Background:**

In 2003 mean cord blood mercury concentrations in pregnant Bermudian women exceeded levels associated with adverse health outcomes in children. The principal mercury source was local fish species. Public health messages were developed suggesting pregnant women reduce consumption of fish species with higher mercury concentrations (e.g. swordfish), substituting species containing lower mercury concentrations, and elevated omega-3 fatty acids (e.g. anchovies). Recent evidence indicates mercury concentrations in Bermuda’s pregnant women have fallen five- fold.

**Objectives:**

Assess whether changes in women’s fish eating patterns during pregnancy are consistent with the public health messaging. Determine who is making changes to their diet during pregnancy and why.

**Methods:**

Mixed methods study with a cross-sectional survey of 121 pregnant women, including 13 opened-ended interviews. Health system, social vulnerability, public health messaging, and socio-demographic variables were characterized and related to changes in fish consumption during pregnancy. Qualitative data were coded according to nutritional advice messages, comprehension of communication strategies, and sources of information.

**Results:**

95% of women surveyed encountered recommendations about fish consumption during pregnancy. 75% reported modifying fish eating behaviors because of recommendations. Principal sources of information about fish consumption in pregnancy were health care providers and the Internet. 71% of women reported reducing consumption of large fish species with greater mercury levels, but 60% reported reduced consumption of smaller, low mercury fish. No participant mentioned hearing about the benefits of fish consumption. More frequent exposure to public health messages during pregnancy was associated with lower reported consumption. Bermudian born women were less likely to reduce consumption of large fish species during pregnancy.

**Conclusions:**

In Bermuda, public health messages advocating reduced consumption of larger, higher mercury-containing fish species appear effective, but masked the nutritional value message of small fish species, with low mercury concentration. Adjustment is needed to better balance the risk communication.

## Introduction

In 2003, our research team, as part of the Atlantis Mobile Laboratory visit to Bermuda, documented elevated mercury concentrations in the cord blood of pregnant women; 60% of samples were above the contemporaneous United States (U.S) Environmental Protection Agency guidelines for mercury in cord blood (30nmol/L or ≈5.8 μg/L) [1]. Eighty-five percent of the mercury analyzed in cord blood was methylmercury, indicating that fish consumption was the primary exposure route [1, 2].

Even at relatively low levels (<20ug/L in cord blood), prenatal exposure to mercury, specifically methylmercury, is associated with childhood impairments in general cognition, memory and verbal skills [3]. Recent research observes associations between prenatal mercury exposure and shortened gestation resulting in reduced newborn birth weight, length and head circumference [4]. In the 2003 study from Bermuda, our research established a mean cord blood mercury concentration of 8.3 μg/l (range 1–32.1 μg/l) [5]. Research from Northern Canada in a population of school-age children, documents adverse effects on behaviour [6] and cognitive function [7]- including intellectual disability [8]- associated with prenatal mercury exposure at concentrations similarly observed in Bermuda in 2003.

According to the Bermuda Department of health, in the early 2000s, dietary recommendations regarding fish consumption were reportedly regularly made to pregnant women in Bermuda, but applied only to imported fish. Bermuda is an archipelago of seven main islands and most inhabitants consume both imported and locally-caught fish species. Dietary surveys completed by the women sampled in 2003 indicated that consumption of certain locally-caught fish species was strongly correlated with cord mercury concentrations, while consumption of imported fish, which were consumed less frequently than local ones, was not related to blood mercury levels [1]. As a result, between 2003 and 2006, the research team assessed levels of mercury, as well as beneficial fish nutrients (omega three fatty acids and selenium) in 307 specimens from 43 commercial fish species caught in Bermudian waters [2]. Omega-3 fatty acids have been associated with reduced risk of preterm birth and greater infant birth-weight [9] and have been linked with enhanced visual system function [10] and memory [11] and language development [12]. Selenium may protect against miscarriage, pre-eclampsia, foetal growth restriction, and preterm labour [13]. In fish eating populations, high selenium and omega-3 intakes may provide health benefits that offset some of the deleterious effects of mercury exposure [14, 15]. It is uncertain if pregnant Bermudian women are aware of these benefits.

For most coastal communities, local fish and seafood continue to contribute to food security and economic prosperity and have substantial cultural and historical importance [16]. Fishing, and the sharing of fish, are important parts of Bermuda’s cultural identity [2]. Consequently, in collaboration with the Government of Bermuda, we developed recommendations for Bermudian pregnant women that incorporated data on mercury, omega-3 fatty acids and selenium concentrations in local fish [2]. For the 43 species for which we had data, we calculated the number of fish portions (220 g) that could be consumed per week by pregnant women following the Food and Agricultural Organization/World Health Organization provisional tolerable weekly intake (PTWI) recommendations for methylmercury (1.6 μg/kg bodyweight)[2]. Based on these calculations, visual communication tools were developed showing fish on a scale according to mercury, selenium and omega-3 fatty acid concentrations. We also made specific recommendations for fish consumption during pregnancy that would help assure that pregnant women did not reach the PTWI [2]. Fish species, such as swordfish and blue marlin, were categorized as “do not consume” during pregnancy, while some were categorized as restricted (e.g. snapper, tuna and Wahoo), and others were grouped as “without restriction,” (e.g. barber and flying fish). We specifically avoided suggesting that women stop eating fish but rather, that they substitute higher-risk fish species (do not consume and restricted categories) with those of less concern and with elevated nutrient profiles (without restriction category) [2]. Following our recommendations, in December 2007, the Department of Health issued local fish consumption guidelines to reduce the exposure of pregnant women to mercury, while also emphasizing the nutritional benefits from regular fish consumption. These were directed specifically to all physicians on the islands [17, 18]. The guidelines were disseminated by local media [19] and continue to be discussed by obstetricians during prenatal consultations. The Department of Health also created a brochure about local fish consumption during pregnancy to accompany more general international guidelines that are discussed during prenatal visits. Despite these efforts, the Department of Health did not know if their messages were reaching pregnant women and if so, how they were being understood.

In 2010, our research team returned to Bermuda and conducted a study on prenatal exposure to environmental contaminants including an analysis of mercury concentrations in pregnant women’s blood. This follow-up study showed that mercury levels in pregnant Bermudian women in 2010 were five fold lower than when first measured in 2003 [17]. None of the participants sampled exceeded international thresholds for concern (8 ug/L) [20] and based on these results, the authors suggested that Bermuda Department of Health guidelines and outreach efforts were effective at reducing the exposure of pregnant women to mercury through consumption of both local and imported fish [17]. However, the survey was carried out at Bermuda’s Public Health Clinic, whose clientele is largely lower income and uninsured, raising questions about whether the results could be generalized to the wider population (e.g. those with high income levels and insurance). While encouraged by the drop in mercury levels in Bermudian pregnant women, it was determined important to develop a fuller understanding of the mechanisms that may have led to this downward shift and to examine the connection between the observed changes in mercury concentrations and Bermuda’s proactive public health activities. In part, the current study is an effort to corroborate, or contradict, the previous study’s conclusions.

Here, we examine whether women are *changing* their fish eating patterns since becoming pregnant and whether public health messages are influencing these behaviors. Second, we document *who* is making changes to their diet. Socio-demographic characteristics such as age, parity, education, income and ethnicity may affect whether women receive and follow dietary recommendations during pregnancy [21, 22]. Finally, based on research from the U.S. that showed an overall decline in fish consumption following a national mercury advisory [23], we were concerned that pregnant women in Bermuda were reducing their consumption of *all* fish, not just those with elevated mercury concentrations. As the aim of the guidelines promoted by the Department of Health were to *balance* the risks and benefits of fish consumption during pregnancy, we assess whether pregnant women are also reducing their consumption of beneficial/low-risk fish species. In other words, if the public health messages were entirely successful, pregnant women would have reduced their consumption of large, steak fish such as swordfish, wahoo, and fresh tuna, while continuing to eat or even increasing their consumption of fish that contain low levels of mercury and are elevated in healthy nutrients [2].

## Materials and Methods

### Context

Bermuda consists of nearly 200 coral islands and islets in close proximity to each other; the landmass stretches approximately 22 miles long and is about 1 mile across. Bermuda is approximately 500 miles off the coast of North Carolina and contains approximately 65,000 permanent residents. It is a self-governing overseas territory of Great Britain, with most people employed in the financial/international business industry, public administration, or the service sector [24]. In 2014, the median gross earnings in Bermuda was 63,897 U.S. dollars [25]. Close to 100% of the population is literate and on average, Bermudians receive 12 years of schooling [24]. The median age of the population is 41, with over a third of the population 45 years or older [24]. The fertility rate in Bermuda is estimated at 2 births per woman; 50% of women have their first child before 25 years of age and nearly a quarter before 20 [24, 26].

### Study design

This is a mixed methods study that employed an explanatory sequential design; that is, the quantitative portion of the study was followed by a qualitative portion to provide context to the quantitative results [27]. First, we conducted a cross-sectional survey with an anonymous, closed-ended questionnaire. At the end of the questionnaire, the participant could opt-in to the qualitative portion by checking a box stating that she would be willing to be contacted later with additional questions. In that box, she provided her contact details (phone number and email address). Those women unwilling to be contacted in the future did not provide personal details and their questionnaires remained anonymous. For those who opted-into the qualitative portion of the study, we carried-out semi-structured interviews and open-ended questionnaires, administered by telephone and email, respectively.

### Quantitative Cross-sectional Study

#### Population Sample

For the quantitative portion of the study, all pregnant women receiving prenatal care on the islands of Bermuda from March 15, 2013 to July 1, 2013, were eligible to participate in this study. All clinical medical practices, private and public, participated in this study. Women were sampled if they were between their 26^th^ and 30^th^ week of pregnancy.

#### Sampling strategy

The questionnaire was self-administered. Women were given the questionnaire by a health service provider during their prenatal glucose challenge appointment. This is a routine test that is a part of every woman’s prenatal care package in Bermuda. It is also a good time for survey administration, because women are required to spend an hour in the clinic/hospital waiting room between blood draws, allowing sufficient time to complete the survey. The questionnaire was distributed towards the end of pregnancy so that public health messages on fish consumption given early in pregnancy could be incorporated into the daily behaviours and practices of pregnant women.

#### Variables of Interest

Covariates: Covariates were divided into four categories: 1) Health system; 2) Social vulnerability; 3) Public health messaging; and 4) Socio-demographic and household.

Health system: For this category, we enquired whether the participant had full medical insurance or partial insurance/no insurance. We also recorded whether the participant was being followed by a private medical provider or a public provider.

Social vulnerability: We enquired whether the participant was receiving any form of government assistance (daycare supplement, financial assistance, or housing assistance). We also administered the 6-item USDA food security module [28]. A participant was considered food insecure if she responded affirmatively to two or more of the questions in the module; otherwise, she was considered food secure. To measure housing security, we used three questions adapted from the Pregnancy Risk Overview questionnaire [29]: 1) In the past 12 months, did you stay in a shelter or other temporary facility for 1 or more nights? (yes/no); 2) In the past 12 months, did you stay with family or friends, as a temporary situation, for 6 months or more? (yes/no); 3) Are you worried about not having a place to live after childbirth? (yes/no). If a participant answered yes to any one of these questions, she was considered housing insecure; otherwise, she was considered housing secure.

Public health messaging: We enquired about communication the participants had received concerning the consumption of fish during pregnancy. Specifically, we asked: 1) Have you ever heard or read any health recommendations about eating fish during pregnancy? (yes/no); 2) While pregnant, how often have you heard or read recommendations about consuming fish? (never, rarely, occasionally, frequently). Because only six women reported that they had never encountered recommendations about consuming fish during pregnancy, we grouped the “never” and “rarely” categories together. We also recorded the sources of this information (health provider, childbirth education class, newspaper, television, internet, friends/family, or other).

Socio-demographic and household characteristics: Data was collected on the participant’s age, civil status, race, place of birth, educational attainment, income, perceived income sufficiency, and pregnancy history.

Outcomes: We had two outcome measures: reduced consumption of large fish (yes/no) and reduced consumption of small fish (yes/no). These reflected Department of Health guidelines advocating for lowered consumption of large fish species and maintenance/augmentation of consumption of smaller, lower mercury species. Those who reduced consumption of large species were in “compliance” with official messages, while those who also reduced small fish were “not in compliance.” To obtain these outcome measures, we asked the following questions: 1) For larger, steak fish such as swordfish, wahoo, and fresh tuna, please check whether you are now consuming a lot less, less, the same, more, or a lot more, *now that you are pregnant*; 2) For smaller fish like anchovies and sardines, please check whether you are now consuming a lot less, less, the same, more, or a lot more, *now that you are pregnant*. The example large fish species we used corresponded to those in the restricted/do not consume categories, while the example small fish species corresponded to the no /limited restriction categories of Bermuda Fish Consumption Guidelines for pregnancy. Those who checked the boxes “a lot less” and “less” were considered to have reduced their consumption of these fish (yes value), while those who checked the boxes “the same, more, or a lot more” were categorized with a no value. For both of these questions, we also had a “do not eat” box.

#### Quality Assurance

The first and second authors drafted the questionnaire. It was reviewed and commented upon by the Chief Medical Officer of Bermuda (fourth author). It was further revised by four maternity staff, including a nurse and nutritionist from the public sector and an obstetrician and pediatrician from the private sector. Prior to beginning the study, staff from the participating clinics were trained on how to distribute the questionnaire and respond to participant questions. The first author and participating clinics communicated regularly and any concerns about questionnaire content and administration were addressed immediately.

#### Statistical Analysis

STATA/SE 13.1 (College Station, Texas) was used to analyze the quantitative data. Bivariate analyses were conducted to assess associations between covariates and outcome variables for those women who eat fish. For categorical data we used two-by-two contingency tables with Fisher’s exact test and for continuous data, we used Student’s t-test. Because the sample is relatively small, we present all covariates with p-values of 0.10 or less.

### Qualitative Study

For all women who agreed to be contacted for the qualitative study (N = 38), we first reached out by phone and then by email. In general, we would make two phone calls and two email attempts before moving on to the next potential participant. Five women were reached by phone for interview, while 7 were communicated with via email. Interview notes were transcribed into Microsoft Word; email correspondence was likewise copied into Word. All textual documents were then uploaded into MAXQDA, a software program for qualitative data analysis. A qualitative codebook was developed based on the study objectives. Data were then coded and grouped into themes of nutritional advice (promotion versus avoidance messages), food communication (satisfactory, dissatisfactory, or confusing), and communication sources. These themes were then related back to the quantitative findings to improve interpretation of these results and their relevance.

### Ethics

The Bermuda Hospital Board Ethics Committee approved this research study.

## Results

### Participant Characteristics

121 women were sampled. Table 1 presents the participant characteristics for the categorical variables. The mean participant age was 31 years (range 16–41). Most women were followed by private obstetricians, had a university education, and an income greater than 6000 dollars per month. Nearly two thirds of women were married or previously married, born in Bermuda, and had already given birth. Half of the sample self-identified as black. Over ten percent of women were food or housing insecure.

**Table 1:** Participant characteristics^1^ ^,^ ^2^.

Characteristic	N	%
**Health System**		
**Source of healthcare**	121	
Public		11.60%
Private		88.40%
**Medical insurance**	120	
Partial/None		21.70%
Full		78.30%
**Social Vulnerability**		
**Governmental Assistance**	120	
Yes		6.70%
No		93.30%
**Food insecure**	119	
Yes		15.10%
No		84.90%
**Housing insecure**	121	
Yes		10.70%
No		89.30%
**Public Health Messaging During Pregnancy**		
**Heard/read recommendations about fish consumption**	119	
Yes		95.00%
No		5.00%
**Frequency fish consumption messages were encountered**	118	
Frequently		11.90%
Occasionally		49.20%
Never/Rarely		39.00%
**Socio-demographic & Household**		
**Civil Status**	121	
Single		33.10%
Married/previously married		66.90%
**Race**	121	
Black		47.10%
White		29.80%
Other		23.10%
**Born in Bermuda**	121	
Yes		61.20%
No		38.80%
**First pregnancy**	120	
Yes		41.70%
No		58.30%
**Educational attainment**	121	
Secondary school or less		28.10%
Technical/vocational school		15.70%
University or more		56.20%
**Monthly Household Income**	110	
≤$2999 U.S.		22.70%
$3000-$5999 U.S.		22.70%
≥$6000 U.S.		54.50%
**Housing**	120	
Family/friends		11.70%
Rent		64.20%
Own		24.20%
**Income Sufficiency**	118	
No personal income		23.90%
Does not meet needs		11.90%
Meets needs suitably		39.00%
Meets needs very well		26.30%

^1)^ Results calculated from the quantitative cross-sectional survey

^2)^ Table only contains data from the categorical variables

### Knowledge about mercury in fish and sources of information

One hundred and thirteen women (95%) had heard or read recommendations about fish consumption during pregnancy. Of these women, 89 (75%) stated that since becoming pregnant they had changed their eating habits because of health recommendations about eating fish during pregnancy. From the qualitative interviews, ten women explicitly stated they received or sought nutritional advice about fish during pregnancy. Five mentioned mercury in fish as a reason for changing fish consumption habits. Three women mentioned specific kinds of fish in relation to mercury.

“They [health care provider] gave me a pamphlet about fish. In this pamphlet, it talked about mercury in fish and which fish to avoid; I remember shark and mackerel” (30 years old, married, university education).

Participants frequently cited advice from healthcare providers and the Internet as sources of information about fish consumption during pregnancy. Of the 113 women who had heard or read health recommendations about fish while pregnant, the primary sources of information were healthcare providers (75% of participants), the Internet (62%), and family and friends (47%). When healthcare providers discussed fish during pregnancy, women interpreted these messages to be about avoidance. In none of the qualitative interviews did participants mention healthcare providers promoting the consumption of fish or discussing a balance between risks and benefits.

“[Did your healthcare provider talk to you about nutrition and diet during pregnancy?] yes…fish and seafood, cold cuts, just as warnings not to eat” (38 years old, married, university education).

The Internet was frequently used to fill in communication gaps left by healthcare providers, but could also be a source of confusion. Family and friends were also used to fill these gaps.

“[regarding satisfaction with nutritional communication from healthcare providers]. I am somewhat satisfied, but I did get more information from other sources, and the OB [obstetrician] probably the least amount of information. Also the information she gave was very basic and nothing more than "eat your vegetables and drink water". Anything specific I often read in books or the internet.” (33 years old, married, university education).

“The nurse just gave a brief introduction to the pamphlet on nutrition during pregnancy which was quite helpful but wasn't enough so I searched online for things that I wanted to have like cooked sushi, shellfish, coffee, decaf and pineapples. The internet gave a few mixed signals because some said I can have the stuff above and some said I can't so I just had the foods in moderation” (32 years old, single, technical school).

“The nurse would explain nutritional advice to a point, why I should eat in a certain manner. If I was not sure about something, I would ask my mother” (16 years old, single, not finished with high school).

### Changes in fish consumption during pregnancy

**Fig 1:**
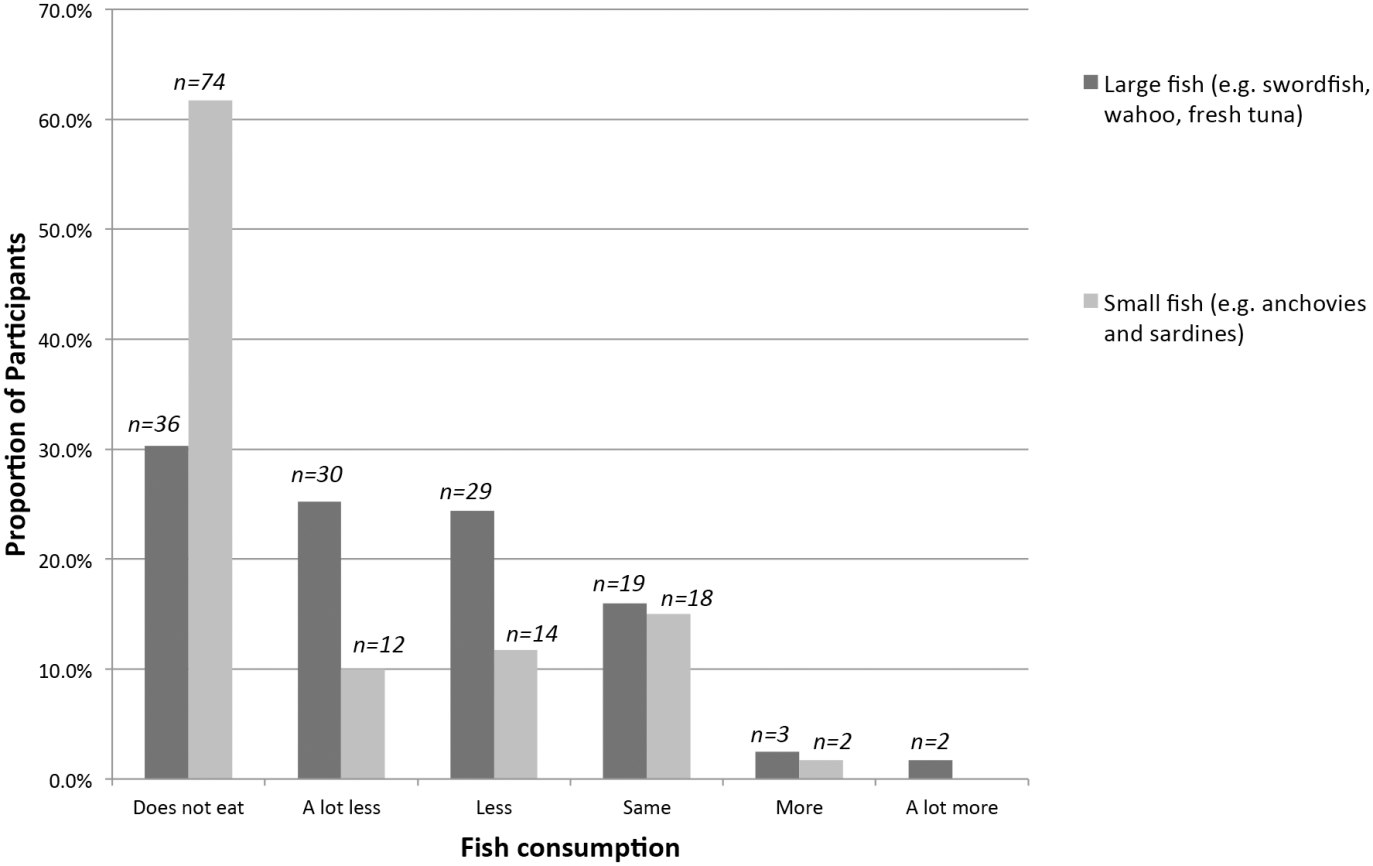
Reported changes in Fish Consumption During Pregnancy (n = 119)^1,2^. 1. Data used to calculate these proportions come from the quantitative surveys. 2. On average, large fish contain more mercury and lower omega-3 and selenium concentrations than smaller fish, which are considered safer to eat.

Fig 1 shows the changes that participants reported having made to their fish consumption during pregnancy. A third of women reported that they did not eat large, steak fish like swordfish, wahoo, and fresh tuna (36, 30%) and most women stated that they did not eat smaller fish like anchovies and sardines (74, 62%). Of those 83 women who ate larger fish, 59 (71%) reported eating less or a lot less fish large fish since becoming pregnant. Four women (5%) reported eating more or a lot more of the large fish. Of those 46 women who ate smaller fish, 22 (57%) reported consuming less or a lot less since becoming pregnant. Two women (4%) reported consuming more small fish since becoming pregnant.

### Reasons given for changing fish consumption

Table 2 depicts the reasons that women gave for reducing their consumption of large and small fish species. For both types of fish, a substantial proportion of women cited advice from a medical professional and the popular press for why they changed their diet. For smaller fish, taste was also an important factor.

**Table 2:** Reasons given by study participants for reducing the consumption of large and small fish species, from among those who reported reduced consumption of these species since becoming pregnant^1^ ^,^ ^2^.

	Large fish species, Proportion of participants	Small fish species, Proportion of participants
Advice from a medical professional	51%	31%
Advice from family or friends	2%	3%
Information in the popular press	37%	23%
Affordability	2%	3%
Does not taste good	13%	37%
Upsets stomach	11%	9%
Other reason	19%	20%

^1.^ Data used to calculate these proportions come from the quantitative surveys.

^2.^ Participants could select more than one reason for reducing consumption of large and small fish species.

### Participant characteristics associated with changes in fish consumption

Table 3 shows characteristics associated with reduced consumption of large fish such as swordfish, wahoo, and fresh tuna. Women followed in the public sector were less likely to reduce their consumption of large fish species, as were women with no medical insurance. It should be noted that lack of insurance is a reason for seeking care in the public sector. None of the social vulnerability variables were associated with reduced consumption of these fish. In regards to public health messaging, all women who reported having frequently heard or read recommendations about fish consumption during pregnancy reduced their consumption of large fish, while 71% of women who occasionally heard such recommendations reduced their consumption, compared to 60% of women who rarely heard such recommendations. Finally, women born in Bermuda and those self-identifying as black, were less likely to reduce their consumption of large fish.

**Table 3:** Characteristics associated with reduced consumption of large fish species during pregnancy, among those women who eat large fish^1^ ^,^ ^2^.

Variable	N	n, % of women who ate less large fish	P-value
**Health System**			
**Source of healthcare**	83		
Private		54, 75.0%	
Public		5, 45.5%	0.07
**Medical insurance**	83		
None		3, 33.3%	
Partial		9, 75.0%	
Full		47, 75.8%	0.04
**Public Health Messaging**			
**Frequency messages about fish consumption were encountered**	81		
Frequently		8, 100%	
Occasionally		27, 71.1%	
Never/Rarely		22, 62.9%	0.05
**Socio-demographic & Household**			
**Race**	83		
Black		25, 59.5%	
White		18, 85.7%	
Other		16, 80.0%	0.07
**Born in Bermuda**	83		
Yes		37, 63.8%	
No		22, 88.0%	0.03

^1.^ Data used to calculate these proportions come from the quantitative surveys.

^2.^ Sample restricted to those who reported that they eat large fish species such as swordfish, wahoo, or fresh tuna

Table 4 shows the categorical characteristics associated with reduced consumption of small fish such as sardines and anchovies. Women with less than a university education were more likely to reduce their consumption of small fish. Younger women were significantly more likely to reduce their consumption of small fish (p 0.03). The mean age of women reporting having reduced their consumption of small fish was 29, compared to 33 for those whose consumption of small fish remained the same. The frequency of encountering messages about fish consumption during pregnancy was positively correlated with a reduction in small fish consumption. Those reporting only “occasionally” encountering fish consumption messages were the least likely to reduce their consumption of smaller fish.

**Table 4:** Characteristics associated with reduced consumption of small fish species during pregnancy, among those women who eat small fish^1^ ^,^ ^2^.

Variable	N	n, %	P-value
**Socio-demographic & Household**			
**Educational attainment**	46		
Secondary school or less		10, 76.9%	
Technical/vocational school		3, 100%	
University or more		13, 43.3%	0.04
**Public Health Messaging**			
**Frequency messages about fish consumption were encountered (N = 78)**	46		
Often		6, 85.7%	
Occasionally		9, 40.9%	
Never/Rarely		11, 64.7%	0.09

^1.^ Data used to calculate these proportions come from the quantitative surveys.

^2.^ Sample restricted to those who eat small fish species such as sardines and anchovies

## Discussion

### Did public health messaging lead to changes in fish consumption during pregnancy?

Public health messaging in Bermuda appears to influence the consumption of fish during pregnancy. Similar findings have been reported in the U.S. and it has been suggested that women may be particularly responsive to behavior change messages during pregnancy [23]. In this study, nearly all women affirmed they had heard or read health recommendations about eating fish during pregnancy and that these led to changes in their fish consumption patterns while pregnant. The qualitative interviews corroborated the quantitative findings; 10 of the 13 women interviewed specifically mentioned fish as part of the nutritional messages they had been exposed to during pregnancy. This study provides support to previous research [17] suggesting that the drop in mercury levels in Bermudian pregnant women between 2003 and 2010 was linked to public health messaging about fish and mercury [17], especially as the majority of women in this study cited their healthcare provider as the source of this information.

### Were certain groups of women more/less likely to change their fish consumption during pregnancy?

Most socio-demographic and household characteristics were not related to changes in fish consumption patterns during pregnancy. However, Bermudian-born and black women were less likely to report reducing their consumption of large fish species. It is important to note significant colinearity between these two variables (p <0.01), as the majority of Bermudian-born women are black (64%). Bermudian-born women may be less likely to reduce their consumption of large fish because of the importance of maritime history to Bermuda and the sense of identity associated with fishing and other activities related to the sea [30]. Moreover, most Bermudians obtain locally-caught fish from family, friends, or small vendors selling on the side of the street. The diversity of such “off-market” fish may be limited and when fish is shared by family and friends, it may be difficult to refuse such generosity or request an alternative lower in mercury. This may be particularly true for lower income women, such as those who use the public health clinic and have no insurance. It may explain, in part, why these women were less likely to report reducing large fish consumption during pregnancy. Alternatively, messaging to women who attend the public health clinic, most of whom do not have insurance, may not sufficiently address mercury concerns in larger fish species, especially if these women have other health concerns that take precedence during the prenatal consultations.

For smaller fish, women with less than a university education and younger women were more likely to report reducing their consumption of these fish. Because education is a proxy for information acquisition and assimilation [31], those with less education may not always fully understand the messages presented to them and/or seek additional information to fill-in communication gaps. In other contexts, researchers have criticized health officials for assuming that awareness of messages about mercury in fish is equivalent to understanding [32].

Concerning larger fish, access to a private healthcare provider may also influence the nutritional advice received by pregnant women. In fact, in correspondence between the first author and the Bermuda Hospital Board, it was revealed that several of the private obstetricians were counseling their patients to reduce all fish intake to one serving per week, irrespective of fish species. Insufficient knowledge about food safety recommendations for pregnant women has been documented elsewhere, as many healthcare professionals receive no formal training on the subject [33]. Health professionals in Bermuda may have insufficient knowledge about the risks and benefits of fish consumption and prefer to take a cautious approach to prenatal counseling that attempts to avoid risk altogether. Previous involvement of healthcare professionals in research about mercury exposure in Bermudian pregnant women may have augmented concerns about the contaminant in fish, which overshadowed the benefits of fish nutrients.

### Did women reduce their consumption of all fish, or just those with high levels of mercury?

Results suggest positive and negative consequences to the public health messaging. Nearly all women reported encountering messages about fish consumption during pregnancy and three-quarters of these women reported modifying their fish consumption patterns based on these messages. From the qualitative interviews, it appears that women were interpreting the messages about fish during pregnancy as being dominated by themes of risk and avoidance. This trend has been noted in wider popular media, where messages about the risks of fish consumption outweigh messages about their benefits, by approximately four to one [34].

The quantitative results showed that among those women who report consuming large steak fish, the vast majority indicated they (71%) had reduced their consumption while pregnant. This finding suggests that messages about avoiding “riskier” fish species are being understood and enacted in Bermuda. However, the nuance in these messages related to the benefits of consuming smaller fish with low levels of mercury, appears to have been received with limited success. Of those women who reported consuming smaller fish, over half also reduced their consumption of these fish while pregnant. Many of these women reported that they were consuming less because of taste, but a nearly equal proportion reported that the change was due to advice from a health professional. Of those who reported reducing small fish consumption because of health professional advice, it is likely that they reduced consumption of all fish species while pregnant (8 of the 10 women who reported consuming less small fish because of advice from a health professional, also reduced consumption of large fish). Of interest, those who reported frequently encountering public health messages about fish in pregnancy were most likely to consume less small fish while pregnant. It is possible that these women were especially risk-adverse and were actively looking up this information. It is also possible that too much exposure to these messages led to a sense of fear about fish altogether, obscuring messaging, if any was received, about the benefits of fish consumption.

### Was it likely that the observed drop in blood mercury levels in Bermudian pregnant women was due to selection from the Public Health Clinic?

Despite the small sample size, it appears that women from the public health clinic were less likely to report changing their fish consumption patterns while pregnant, compared to women followed in the private sector. Women without private insurance were significantly less likely to reduce their consumption of large fish (p 0.04) and women followed at the Public Health Clinic were marginally less likely to report doing so (p 0.07). Given that women followed in the private sector were more likely to reduce their consumption of fish species known to be elevated in mercury, it appears unlikely that the large drop in blood mercury levels between 2003 and 2010 [17] was due solely to selection bias.

### Strengths and Limitations

There are limitations to this study. First, changes in fish consumption are self-reported and thus represent perceived changes, which may not reflect actual change. We did not measure actual fish consumption levels (e.g. mean number of grams consumed per day) and while methods, such as a food frequency questionnaires could have been used to obtain this information they are ill adapted to self-administration and substantially increase the time necessary to complete the interview. Thus, we could not compare actual fish consumption levels in Bermudian women with international standards for the recommended number of grams consumed per week. However, our objective was to investigate whether public health messages were influencing behavior *change* during pregnancy and not to assess dietary consumption patterns. Additionally, because of the small population of Bermuda (≈65,000) and low birth rate, we had a relatively small sample size for the quantitative estimates, which prohibited multivariate analyses, especially as most variables of interest were categorical. Given our objective to describe the characteristics of those making changes to their diets, multivariate analyses would have added little additional information to our findings, as this objective was not etiological.

There are strengths to this study. Unlike previous research on the subject [23], we used a mixed-methods study design, which helped us to better understand why reported changes may have occurred and to identify shortcomings in the public health messages. The qualitative results helped to identify sources of confusion in messages about fish and pregnancy and a perceived general lack of discussion about the benefits of fish consumption from Bermudian healthcare providers. Additionally, we obtained a representative sample of all pregnant women on the island during the sampling period, with a nearly 100% participation rate (we had 2 refusals from the public health clinic). Characteristics of the study sample including race, marital status, and income levels closely resemble those obtained by the 2010 Bermudian census [24], suggesting that study findings can be generalized to all pregnant women in Bermuda. This research program also has the added benefit of being carried in a small geographically isolated community, with an advanced public health system and within the context of several rigorous studies on local fish, from which we have accurate data on the levels of mercury and nutrients.

For future research on health messaging during pregnancy, we suggest examining women’s perceptions about fish/seafood early and late in pregnancy, in order to document the assimilation of prenatal messages across pregnancy and whether changes in fish consumption differ with more exposure to prenatal counseling. We suggest combining such research with biomarker measures of nutrients and contaminants. This study assumes that reductions in fish consumption, especially smaller fish, reduce fetal exposure to beneficial omega-3 fatty acids. However, in the absence of blood measures of these nutrients, our assumption is conjecture. For example, it is possible that women take fish oil supplements to offset their lowered consumption of smaller, oily fish. In this study, of the 84 (69%) women who reported taking prenatal vitamins, 15 (18%) of these women reported taking some form of fish oil. On the other hand, 37 (31%) women did not report taking any prenatal supplements at all.

## Conclusions

This study provides evidence that public health messages by the Government of Bermuda, which advocated that pregnant women consume fewer large fish, may be effective. The majority of women who consume fish report that they eat less large fish species, such as swordfish. Consumption of small fish species with highly beneficial nutrient profiles is reportedly low in this population. Some women reported lowering their consumption of these species during pregnancy, and if these are the only fish they eat, messaging could be unduly adverse to the nutrition of these women. Public health messages may need clarification so that women understand that it is acceptable to continue, even increase, their consumption of smaller fish species during pregnancy. Given the importance of maritime culture to Bermuda, health promotion activities centered on fish or fishing, such as cooking demonstrations with anchovies and sardines, or community-based programs that subsidize or give-away smaller fish species to pregnant women, may be promising avenues of intervention in the future.

## References

[1] Dewailly E, Pereg D, Knap A, Rouja P, Galvin J, Owen R. Exposure and effects of seafood borne contaminants in maritime populations In: Walsh PJ, Smith SL, Fleming LE, Solo-Gabriele H, Gerwick WH, editors. Ocean and Human Health: Risks and Remedies from the Seas. Burlington, MA: Academic Press; 2007 p. 670.

[2] Dewailly E, Rouja P, Dallaire R, Pereg D, Tucker T, Ward J, et al Balancing the risks and the benefits of local fish consumption in Bermuda. Food additives & contaminants Part A, Chemistry, analysis, control, exposure & risk assessment. 2008;25:1328–38. 10.1080/02652030802175285 19680840

[3] Karagas MR, Choi AL, Oken E, Horvat M, Schoeny R, Kamai E, et al Evidence on the human health effects of low-level methylmercury exposure. Environ Health Perspect. 2012;120:799–806. 10.1289/ehp.1104494 22275730 PMC3385440

[4] Dallaire R, Dewailly E, Ayotte P, Forget-Dubois N, Jacobson SW, Jacobson JL, et al Exposure to organochlorines and mercury through fish and marine mammal consumption: associations with growth and duration of gestation among Inuit newborns. Environment International. 2013;54:85–91. 10.1016/j.envint.2013.01.013 23422685 PMC3632409

[5] Baird J, Cooper C, Margetts BM, Barker M, Inskip HM. Changing health behaviour of young women from disadvantaged backgrounds: evidence from systematic reviews. Proceedings of the Nutrition Society. 2009;68:195–204. 10.1017/S0029665109001050 19208272

[6] Boucher O, Jacobson SW, Plusquellec P, Dewailly E, Ayotte P, Forget-Dubois N, et al Prenatal methylmercury, postnatal lead exposure, and evidence of attention deficit/hyperactivity disorder among Inuit children in Arctic Quebec. Environ Health Perspect. 2012;120:1456–61. 10.1289/ehp.1204976 23008274 PMC3491943

[7] Boucher O, Muckle G, Jacobson JL, Carter RC, Kaplan-Estrin M, Ayotte P, et al Domain-specific effects of prenatal exposure to PCBs, mercury, and lead on infant cognition: results from the Environmental Contaminants and Child Development Study in Nunavik. Environ Health Perspect. 2014;122:310–6. 10.1289/ehp.1206323 24441767 PMC3948023

[8] Jacobson JL, Muckle G, Ayotte P, Dewailly E, Jacobson SW. Relation of Prenatal Methylmercury Exposure from Environmental Sources to Childhood IQ. Environ Health Perspect. 2015; 123: 827–33. 10.1289/ehp.1408554 25757069 PMC4529008

[9] Imhoff-Kunsch B, Briggs V, Goldenberg T, Ramakrishnan U. Effect of n-3 long-chain polyunsaturated fatty acid intake during pregnancy on maternal, infant, and child health outcomes: a systematic review. Paediatric and Perinatal Epidemiology. 2012;26 Suppl 1:91–107. 10.1111/j.1365-3016.2012.01292.x 22742604

[10] Jacques C, Levy E, Muckle G, Jacobson SW, Bastien C, Dewailly E, et al Long-term effects of prenatal omega-3 fatty acid intake on visual function in school-age children. The Journal of Pediatrics. 2011;158:83–90. 10.1016/j.jpeds.2010.06.056 20797725 PMC2992831

[11] Boucher O, Burden MJ, Muckle G, Saint-Amour D, Ayotte P, Dewailly E, et al Neurophysiologic and neurobehavioral evidence of beneficial effects of prenatal omega-3 fatty acid intake on memory function at school age. The American Journal of Clinical Nutrition. 2011;93:1025–37. 10.3945/ajcn.110.000323 21389181 PMC3076654

[12] Strain JJ, Davidson PW, Thurston SW, Harrington D, Mulhern MS, McAfee AJ, et al Maternal PUFA status but not prenatal methylmercury exposure is associated with children's language functions at age five years in the Seychelles. J Nutr. 2012;142:1943–9. 10.3945/jn.112.163493 23014496 PMC3498972

[13] Mistry HD, Broughton Pipkin F, Redman CW, Poston L. Selenium in reproductive health. American journal of obstetrics and gynecology. 2012;206:21–30. 10.1016/j.ajog.2011.07.034 21963101

[14] Choi AL, Mogensen UB, Bjerve KS, Debes F, Weihe P, Grandjean P, et al Negative confounding by essential fatty acids in methylmercury neurotoxicity associations. Neurotoxicology and teratology. 2014;42:85–92. 10.1016/j.ntt.2014.02.003 24561639 PMC4051703

[15] Lemire M, Fillion M, Frenette B, Mayer A, Philibert A, Passos CJ, et al Selenium and mercury in the Brazilian Amazon: opposing influences on age-related cataracts. Environ Health Perspect. 2010;118:1584–9. 10.1289/ehp.0901284 20716509 PMC2974697

[16] FAO. World Food Summit. Rome: 1996.

[17] Dewailly E, Rouja P, Forde M, Peek-Ball C, Cote S, Smith E, et al Evaluation of a public health intervention to lower mercury exposure from fish consumption in bermuda. PloS one. 2012;7(10):e47388 10.1371/journal.pone.0047388 23077607 PMC3471810

[18] Department of Health. Risks and Benefits of Local Fish Consumption- Advisory. Hamilton, Bermuda: 2007.

[19] Smith T. Mercury concern for some local fish. The Royal Gazette. 2007.

[20] Legrand M, Feeley M, Tikhonov C, Schoen D, Li-Muller A. Methylmercury blood guidance values for Canada. Can J Public Health. 2010;101:28–31. 20364534 10.1007/BF03405557 PMC6973950

[21] Mallard SR, Houghton LA. Public health policy to redress iodine insufficiency in pregnant women may widen sociodemographic disparities. Public Health Nutr. 2014;17:1421–9. 10.1017/S1368980013001626 23777645 PMC10282430

[22] Ray JG, Singh G, Burrows RF. Evidence for suboptimal use of periconceptional folic acid supplements globally. BJOG: an international journal of obstetrics and gynaecology. 2004;111: 399–408. 15104602 10.1111/j.1471-0528.2004.00115.x

[23] Oken E, Kleinman KP, Berland WE, Simon SR, Rich-Edwards JW, Gillman MW. Decline in fish consumption among pregnant women after a national mercury advisory. Obstetrics and gynecology. 2003;102:346–51. 12907111 10.1016/S0029-7844(03)00484-8 PMC1989666

[24] Department of Statistics. 2010 Census of Population & Housing Final Results. 2011.

[25] Department of Statistics. Labour Market Indicators. Hamilton, Bermuda: Government of Bermuda, 2015.

[26] The World Bank. World Development Indicators [cited 2015 June 26]. Available from: http://databank.worldbank.org/data//reports.aspx?source=2&country=BMU&series=&period=.

[27] Creswell J, Plano Clark V. Designing and Conducting Mixed Methods Research, 2nd edition Thousand Oaks, California, USA: Sage; 2011.

[28] Bickel G, Nord M, Hamilton W, Cook J. Guide to Measuring Household Food Security. Revised 2000.

[29] Harrison PA, Sidebottom AC. Systematic prenatal screening for psychosocial risks. Journal of Health Care for the Poor and Underserved. 2008;19:258–76. 10.1353/hpu.2008.0003 18264001

[30] Andrews C. Conceptualizing heritage through the maritime lens: A heritage ethnography of maritime Bermuda. International Journal of Heritage Studies. 2012;18:352–68.

[31] Shimshack JP, Ward MB, Beatty TKM. Mercury advisories: Information, education and fish consumption. Journal of Economics and Management. 2007;53:158–79.

[32] Furgal C, Powell S, Myer H. Digesting the message about contaminants and country foods in the Canadian North: A review and recommendations for future research and action. Arctic 2005;58:103–14.

[33] Morales S, Kendall PA, Medeiros LC, Hillers V, Schroeder M. Health care providers' attitudes toward current food safety recommendations for pregnant women. Appl Nurs Res. 2004;17:178–86. 15343551 10.1016/j.apnr.2004.06.006

[34] Greiner A, Clegg Smith K, Guallar E. Something fishy? News media presentation of complex health issues related to fish consumption guidelines. Public Health Nutr. 2010;13:1786–94. 10.1017/S1368980010000923 20519047

